# Specific EEG Encephalopathy Pattern in SARS-CoV-2 Patients

**DOI:** 10.3390/jcm9051545

**Published:** 2020-05-20

**Authors:** Jesús Pastor, Lorena Vega-Zelaya, Elena Martín Abad

**Affiliations:** 1 Clinical Neurophysiology and Instituto de Investigación Biomédica, Hospital Universitario de La Princesa, C/Diego de León 62, 28006 Madrid, Spain; lorenacarolina.vega@salud.madrid.org; 2 Clinical Neurophysiology, Hospital Universitario de La Princesa, C/Diego de León 62, 28006 Madrid, Spain; emabad@salud.madrid.org

**Keywords:** cardiorespiratory arrest, correlation coefficient, fast fourier transform, quantified EEG, spectral entropy

## Abstract

We used quantified electroencephalography (qEEG) to define the features of encephalopathy in patients released from the intensive care unit after severe illness from COVID-19. Artifact-free 120–300 s epoch lengths were visually identified and divided into 1 s windows with 10% overlap. Differential channels were grouped by frontal, parieto-occipital, and temporal lobes. For every channel and window, the power spectrum was calculated and used to compute the area for delta (0–4 Hz), theta (4–8 Hz), alpha (8–13 Hz), and beta (13–30 Hz) bands. Furthermore, Shannon’s spectral entropy (SSE) and synchronization by Pearson’s correlation coefficient (*ρ*) were computed; cases of patients diagnosed with either infectious toxic encephalopathy (ENC) or post-cardiorespiratory arrest (CRA) encephalopathy were used for comparison. Visual inspection of EEGs of COVID patients showed a near-physiological pattern with scarce anomalies. The distribution of EEG bands was different for the three groups, with COVID midway between distributions of ENC and CRA; specifically, temporal lobes showed different distribution for EEG bands in COVID patients. Besides, SSE was higher and hemispheric connectivity lower for COVID. We objectively identified some numerical EEG features in severely ill COVID patients that can allow positive diagnosis of this encephalopathy.

## 1. Introduction

Severe acute respiratory syndrome coronavirus 2 (SARS-CoV2) causes an acute, highly lethal disease, COVID-19. This disease was first detected in December 2019 in China and rapidly spread around the world. Neurological complications in COVID-19-infected patients have been reported. Reported central nervous system (CNS) effects include encephalitis [1,2], toxic encephalopathy (ENC) [3,4,5], dysgeusia and anosmia [6], and acute cerebrovascular disease [7,8]. The mechanisms of CNS infection and its pathophysiology by CoV2 are still debated, and direct invasion through the blood–brain barrier (BBB), a neuronal pathway, hypoxic damage, immune-response mediated injury, and angiotensin-converter enzyme 2, among others, have been proposed [3,9].

Encephalopathy, also called altered mental status or confusional state, refers clinically, descriptively, or pathologically to a state of impaired cognition, generally acute or subacute [10]. Acute or infectious toxic encephalopathy refers to a type of reversible brain syndrome caused by several factors, such as a metabolic disorder, systemic toxemia, or hypoxia during an acute infection [11,12,13,14]. The pathological changes include cerebral edema, with no evidence of inflammation in cerebrospinal fluid. Symptoms are complex and diverse. The electroencephalogram (EEG) defined in [15] is similar to that described in one case of COVID-19, showing bilateral and focal slowing in the left temporal region with sharp waves (see [4]). Descriptions of EEG are based on classical analysis by visual inspection. In recent years, however, the development of mathematical analysis tools for bioelectric signals, commonly known as quantified EEG (qEEG), has introduced elements of objectivity into the analysis of EEG records [16,17]. With this goal in mind, we developed a qEEG using classical mathematical methods, but in a neurophysiologically and clinically oriented fashion. We started from the assumption that EEG is based in a homeostatic system [18,19], in order to establish an approximate direct relationship between variations in numerical magnitude and the underlying anatomo-functional system.

We applied this method of qEEG to patients discharged from the intensive care unit (ICU) after COVID-19. We used two control groups from patients previously studied in our hospital: (i) patients with infectious toxic encephalopathy, taken as a kind of gold-standard encephalopathic pattern, and (ii) patients after cardiorespiratory arrest, as an example of severe hypoxic insult to the CNS. The main aim of our work is to describe the structure of EEG for the three groups of patients, especially the COVID group. This structure is defined by the relative distribution of different bands (δ, θ, α, β) across the brain lobes and by global synchronization, defined at the lobe and hemisphere level. We hypothesize that these structures can be specific, to some degree, for every pathological state.

## 2. Materials and Methods

### 2.1. Patients and Definitions

We analyzed scalp EEGs performed in 20 patients (17 men, 3 women) diagnosed with COVID-19 after a stay of several days and following release from the ICU. Recordings were performed while patients were hospitalized but in the non-critical care unit and were required because patients had shown clinical alterations of awareness or cognitive state. Patients were over 18 years old and their medical history was collected (see Appendix A, Table A1, Table A2 and Table A3). The experimental procedure was approved by the medical ethical review board of the Hospital Universitario de La Princesa and deemed “care as usual.” Under these circumstances, written informed consent was not required.

We used two control groups to try to identify possible pathophysiological mechanisms. Both groups were obtained from previously studied hospitalized patients and were defined as follows: (i) to evaluate the effect of severe hypoxemia, we assessed patients who underwent resuscitation after cardiorespiratory arrest (CRA; 20 men and 1 woman), and (ii) we assessed inpatients evaluated with mild/moderate cognitive impairment showing encephalopathy (ENC) on EEG (16 men and 15 women). In both cases, recordings showing intense irritative activity or patients with focal/localized pathology (e.g., craniectomy, previous surgery, cerebrovascular disease) were excluded.

Patients were clinically classified as fully alert, confused, stuporous, or comatose. To obtain a global clinical state for the group, we computed the weighted mean, assigning a range from 1 (fully alert) to 4 (coma). A value near 1 indicates a less clinically affected group, and a value near 4 indicates a majority of patients in coma, thus severely affected.

We visually analyzed the EEGs according to the definitions of the American Clinical Neurophysiology Society’s Standardized EEG Terminology [20,21]. Visually analyzed encephalopathy patterns have been categorized in 4 grades: (i) grade I, excess of slow posterior activity; (ii) grade II, predominant theta activity in more than 50% of recording; (iii) grade III, predominant delta activity in more than 50% of recording; and (iv) grade IV, burst-suppression pattern [10,22].

### 2.2. Electroencephalogram (EEG) Recording

EEG recordings were performed always in the basal state of patients using a 32-channel digital system (EEG32U, NeuroWorks, XLTEK^®^, Oakville, ON, Canada) with 19 electrodes placed according to a 10–20 international system. In addition, Einthoven’s differential derivation I for ECG was placed. Recordings were performed at a 512 Hz sampling rate, with a filter bandwidth of 0.5 to 70 Hz and notch filter of 50 Hz. Electrode impedance was usually below 15 kΩ.

Clinical reports were prepared by a clinical neurophysiologist with several years of experience in electroencephalography. qEEG was performed online to facilitate better information of EEG status.

Voltage amplitude (in µV) was obtained as the root mean square of occipitoparietal differential channels. The same channels were used to identify the dominant frequency of posterior rhythm, defined as the highest value of the power spectrum above 4 Hz. In some patients, just the delta component was observed, and its maximum value was chosen as the dominant posterior rhythm.

Artifact-free periods (excluding electro-oculogram or muscular movement in awake patients) were selected and exported in ASCII files to be quantified.

### 2.3. Quantification of EEG

**Figure 1:**
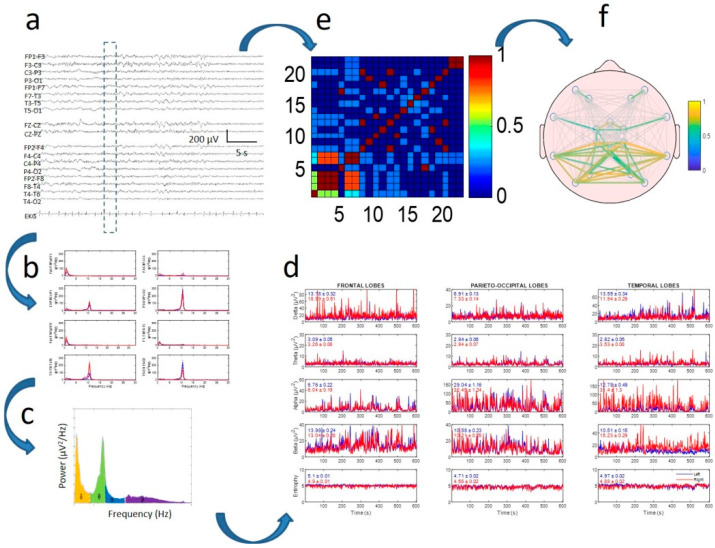
Method of electroencephalogram (EEG) quantification in two branches: power spectra (**b**–**d**) and synchronization (**e**,**f**). (**a**) Raw EEG tracing. Discontinuous rectangle shows moving window used for analysis; (**b**) power spectra for every channel; (**c**) areas for delta, theta, alpha, and beta bands under spectrum highlighted in different colors; (**d**) dynamics of four bands (and entropy in lower row) for every lobe. Mean and standard error of the mean (SEM) values for every tracing are displayed inside every graph. Red and blue lines indicate right and left hemispheres, respectively; (**e**) correlation matrix for window; (**f**) mean correlation computed for all recordings.

The algorithm used was previously published [17]. Classical EEG bands used in the analysis were defined as delta (δ) = 0.5–4.0, theta (θ) = 4.0–8.0, alpha (α) = 8.0–13.0, and beta (β) = 13.0–30.0 Hz (see Appendix B for more details). The process is indicated in Figure 1. All records were between 120 and 300 s, which allowed a minimum of 130 and a maximum of 330 windows to be computed.

Numerical analysis of EEG recordings was performed with custom-made MATLAB^®^ R2019 software (MathWorks, Natick, MA, USA).

### 2.4. Statistics

EEG bands were normalized to the whole spectrum, therefore values are given in percentages.

Statistical comparisons between groups were performed using Student’s *t*-test or analysis of variance (ANOVA) for data with normal distribution. Normality was evaluated using the Kolmogorov–Smirnov test. Mann–Whitney rank sum test or ANOVA on ranks was used when normality failed. In the last case, either the Tukey or Holms–Sidak test was used for all pairwise post hoc comparisons of mean ranks of groups. Chi-square test ($\chi^{2}$) was used to assess the differences in EEG bands between groups of patients. This test cannot be used when numerical values are lower to unity, therefore comparison for Shannon’s spectral entropy (SSE) and correlation was done with values normalized to ENC patients. SigmaStat^®^ 3.5 software (SigmaStat, Point Richmond, CA, USA) and MATLAB^®^ were employed for statistical analysis.

The significance level was set at *p* = 0.05. Results are shown as mean ± standard error of the mean (SEM), except where otherwise indicated.

## 3. Results

### 3.1. Patients

The main clinical features of the three groups can be seen in Table A1, Table A2 and Table A3 in Appendix A. All COVID patients showed severe illness (including pneumonia, desaturation, and inflammatory systemic response), indicating a need for intubation and sedo-analgesia in the ICU for several days [23]. Two patients were fully alert, 10 were mildly to moderately confused, and eight stuporous. Bearing in mind the small number of patients, we did not separately analyze recordings from stuporous and confused patients, considering them all as different degrees of the same continuum. Irritative activity was observed in four patients.

Overall, CRA patients were the most severely affected, with 3.7 ± 0.2 (*p* < 0.001, ANOVA on ranks) on the global clinical scale, and severity was similar for COVID and ENC groups (2.3 ± 0.1 and 2.3 ± 0.2 respectively). In spite of the severity, the mean period of hospital admission was similar for all three groups (11.6 ± 3.6, 13.4 ± 2.1, and 8.6 ± 3.6 days for ENC, COVID, and CRA, respectively, ANOVA on ranks). However, the ENC group was older (74.2 ± 3.0 years, *p* = 0.002, ANOVA on ranks) than the COVID and CRA groups (63.9 ± 2.7 and 62.8 ± 2.5, respectively).

Although the minimum values of capillary oxygen saturation (SaO_2_) in COVID patients reached 78%, mean saturation was above 89% (overall group 94.8 ± 0.6%).

### 3.2. Visual Properties of EEG

**Figure 2:**
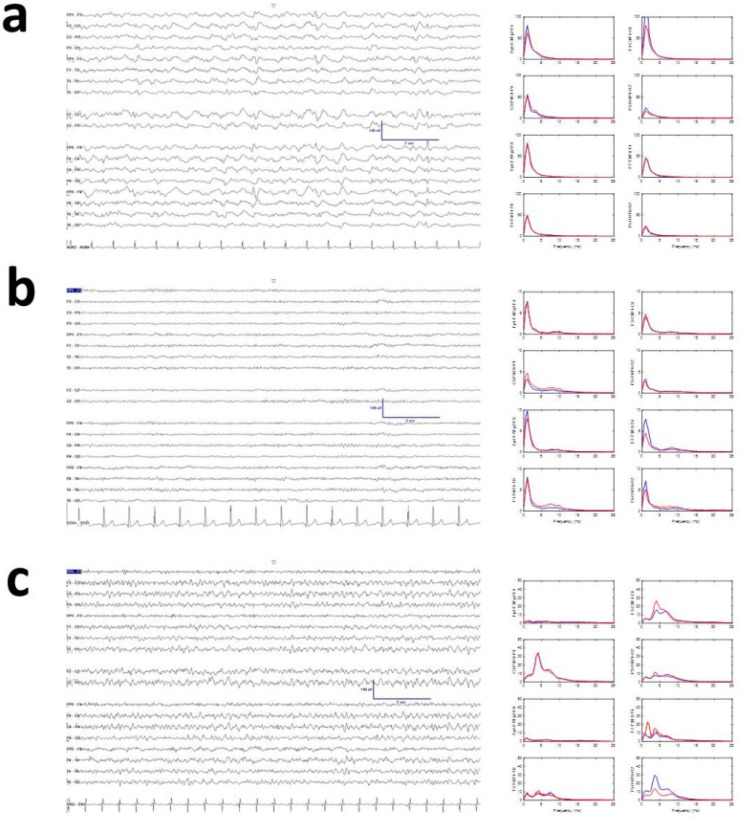
Examples of raw recordings: (**a**) encephalopathy (ENC), (**b**) COVID, and (**c**) cardiorespiratory arrest (CRA). Right column shows mean spectra for channels. Red and blue lines indicate right and left hemispheres, respectively. Y-axis units in µV^2^/Hz.

**Figure A1:**
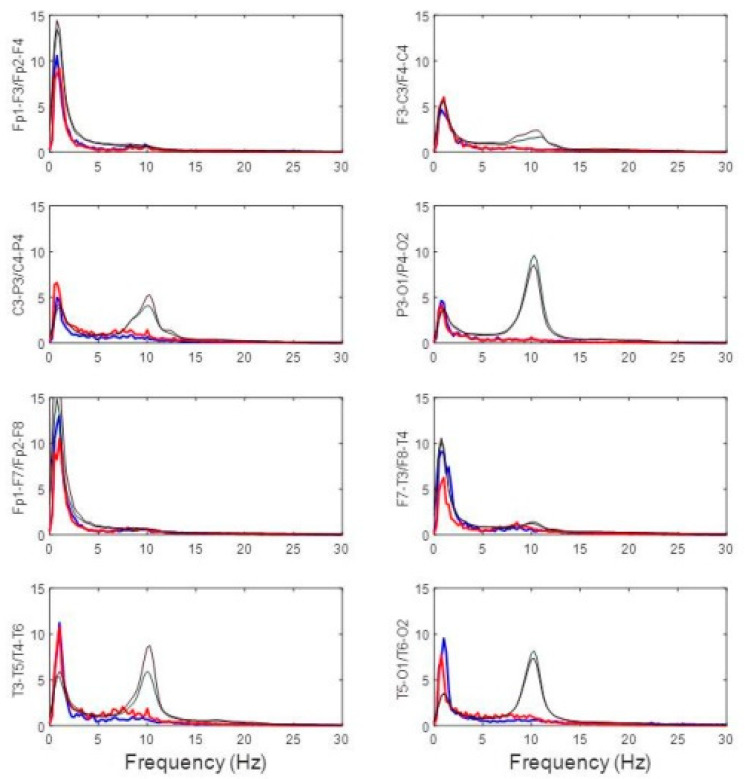
Comparison of mean power spectrum for channels between a COVID patient (#8) and the mean spectra pooled from 30 healthy volunteers, normalized to the maximum amplitude of delta band at central regions. Red and blue lines indicate right and left hemispheres for the COVID patient respectively and black lines represent the volunteers group. Y-axis units in µV^2^/Hz. Pay attention to the great difference for alfa/beta bands.

EEG records of COVID patients showed mean voltage and posterior dominant rhythm in the theta band (17.5 ± 1.4 µV and 6.5 ± 0.8 Hz), with ENC showing a tendency toward higher amplitude (not significant) and slower posterior component (24.3 ± 1.4 µV and 3.8 ± 0.6 Hz). Patients who had experienced CRA showed amplitude similar to COVID patients and frequency similar to ENC patients (17.1 ± 2.4 µV and 4.0 ± 0.4 Hz). Only mean frequency for COVID patients was different (*p* < 0.05, ANOVA on ranks); amplitude was similar for all three groups. Visually, COVID EEG records had less delta activity than infectious toxic encephalopathy, although this turned out to be a false impression when qEEG was performed (see below). Overall, the apparent absence of delta/theta activity conferred a near-physiological aspect to the recordings. Despite the different visual aspects appearing between ENC and COVID, mean spectra by channels were quite similar. Although not analyzed in detail because a comparison with physiological states was out of the scope of this paper, the power spectrum of COVID patients was completely non-physiological (Appendix A, Figure A1). Examples of typical recordings are shown in Figure 2. Another interesting property was the scarce presence of sharp elements (sharp waves or spikes). In fact, we observed sharp waves in EEGs of only 4/20 patients.

### 3.3. Topographic Distribution of EEG Bands

Firstly, we assessed the similarities in the EEG structure, defined by the relative distribution of bands across the scalp. Results are shown in Table 1.

From this table we can observe that EEG structure is truly different for the three groups and for all bands (except for ENC/COVID delta and theta bands, which are similar). Another interesting observation is that the value of $\chi^{2}$ is always greater for ENC/CRA, with the COVID value in the middle of the two groups. Therefore, the structure of the EEG for COVID patients is between the two extreme conditions.

**Figure 3:**
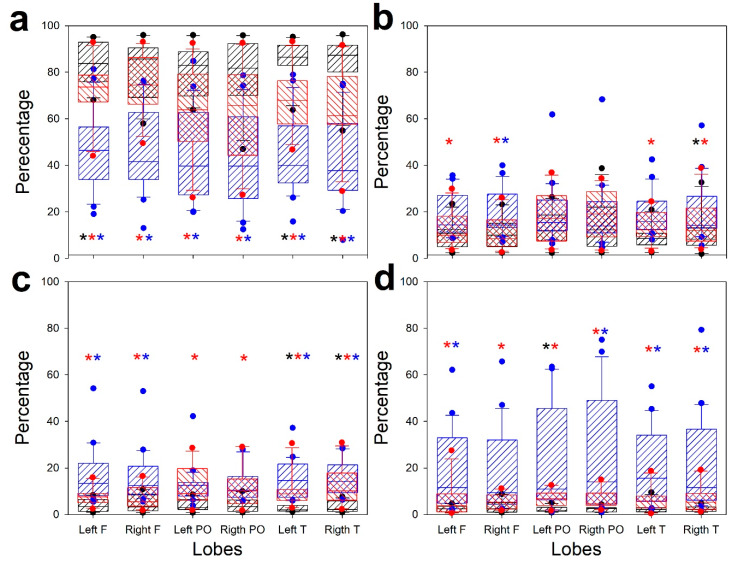
Box plots showing comparison of EEG structure for different bands: (**a**) delta, (**b**) theta, (**c**) alpha, and (**d**) beta. Striped black box: ECN; striped red box: COVID; striped blue box: CRA; black asterisk: difference between ENC and COVID; red asterisk: difference between ENC and CRA; blue asterisk: difference between COVID and CRA.

However, although overall the three groups were dissimilar, we assessed exactly what the differences were for every band. Then, we computed one-way ANOVA (ANOVA on ranks when normality failed) for every lobe and band. These results are shown in Figure 3.

Significant difference above *p* < 0.05 by ANOVA is indicated by asterisks. The post hoc results indicating pairs of groups are presented in a color code. The pattern of ENC and CRA is clearly different for all bands, except for the parieto-occipital theta band. However, the COVID group is not completely different from ENC and CRA, although it is clearly observed that the distribution is between the two extreme groups. Nonetheless, the behavior of temporal lobes clearly differs for ENC and COVID groups for δ, α, and β bands.

### 3.4. Synchronization and Spectral Entropy of EEG

We computed mean synchronization, measured as Pearson’s correlation coefficient (*ρ*), at hemispheric and lobar levels. We also used a measure of the complexity of spectra, SSE. As stated above, global comparison using $\chi^{2}$ was done on values for *ρ* and SSE normalized to ENC. The results are shown in Table 2.

From this table we can observe that the structure of SSE is quite different for the COVID group. However, contrary to what was previously observed, the value of $\chi^{2}$ was greater for ENC/COVID, with the CRA value in the middle of the two groups. However, no differences have been observed for the overall synchronization.

**Figure 4:**
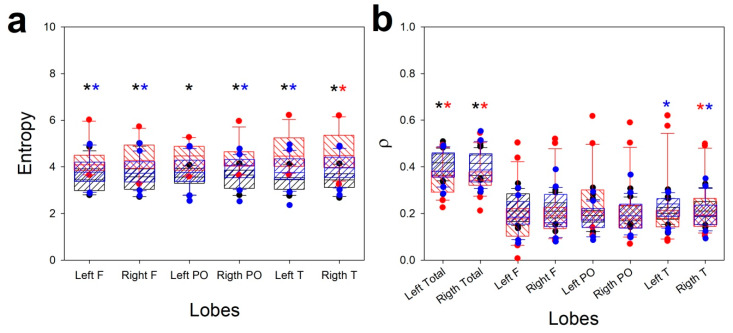
Box plots showing comparison of the structure of entropy and synchronization for different groups: (**a**) Se; (**b**) *p*. Striped black box: ECN; striped red box, COVID; striped blue box, CRA; black asterisk: difference between ENC and COVID; red asterisk: difference between ENC and CRA; blue asterisk: difference between COVID and CRA.

As we did with the bands, we plotted these properties, comparing the three groups for every lobe (and hemisphere, in the case of *ρ*), using ANOVA/ANOVA on ranks (Figure 4).

Contrary to EEG bands, SSE was higher for the COVID group and lower for the ENC group. From Figure 4a we can observe that SSE was always different for COVID from both other groups at all lobes. This result may be surprising considering the kind of spectra shown in Figure 2c, where the distribution is apparently more complex. However, the presence of α and β bands (scarcely present in the CRA group) increased the SSE for COVID patients.

Finally, although *ρ* was not as different between groups as SSE, a clear difference was seen in hemispheric synchronization and temporal lobes for CRA and COVID patients, with lower synchronization for the first group.

## 4. Discussion

We show in this paper that qEEG can differentiate between types of encephalopathies. In this sense, we show that COVID patients display structures of EEG truly distinguishable from infectious toxic encephalopathy on the one hand, and from encephalopathies of patients who experienced a severe hypoxic condition (CRA) on the other. This is the first report of a specific EEG pattern in COVID patients exhibiting encephalopathy and is different from the case report previously published, which shared the same features as infectious and toxic encephalopathy [4]. Obviously, we cannot exclude that this pattern appears in COVID patients, but until now we have not observed this pattern in our patients.

From a methodological point of view, it is quite obvious that the groups do not exactly overlap. Patients from the ENC group were older than those in the COVID and CRA groups. Infectious toxic encephalopathy is not an age-related entity; therefore, it does not seem probable that the difference with COVID patients would be due to this factor. The other group, including patients after severe hypoxia (CRA), was also different, mainly in clinical impairment. We analyzed these patients, because one possible pathophysiological mechanism of nervous injury would be hypoxia [24]. Significantly, the EEG pattern of COVID patients was between those of the ENC and CRA groups. Therefore, it can be speculated that hypoxia may have some participation in this electroclinical entity. In fact, peaks of low saturation were observed in all patients. However, the EEG structures of the CRA and COVID groups were different enough to consider that other factors besides hypoxia must be responsible for the bioelectrical pattern. Nevertheless, the different history of previous medical conditions can influence the brain response. Nonetheless, the presence of previous chronic pathologies was lower for COVID patients (12/20) than for ENC (22/31) or CRA groups (19/21).

Post-intensive care syndrome (PICS) is a complex clinical situation observed in patients discharged from the ICU after a prolonged period of intubation under sedo-analgesia and includes mobility problems of neuromuscular origin, altered cognition, and the development of psychotic manifestations [25]. Risk factors for this syndrome include female sex, older age, previous mental health problems, disease severity, and delirium [26]. To the authors’ knowledge, no reports of encephalopathy in the context of this syndrome have been described. Therefore, although we cannot firmly exclude this possibility as a cause of COVID encephalopathy, neither can we consider it as the first option.

For most of the COVID patients, the raw EEGs show a nearly physiological pattern. However, the mean spectra show the existence of a significant encephalopathic pattern with an excess of generalized delta activity and lower alpha and beta values. Other specific anomalies were also observed. First, SSE, a measure to characterize spectral complexity [27], was higher than in the ENC and CRA groups. We can conclude that the distribution of bands into spectra was more complex for COVID patients’ EEGs, with higher relative amounts of faster bands (α and β). Second, the synchronization was different for COVID patients’ EEGs, showing lower hemispheric values for all three groups. Although it is not proved, we can hypothesize that low hemispheric synchronization may be causally related to the clinical state of confusion observed in all patients, especially those with great discrepancies between nearly normal EEG tracings and cognitive/behavioral alteration. The method used to assess synchronization (*ρ*) has shortcomings deriving from volume conduction [28]. To minimize this problem, we used differential montage, instead of referential [29]. Besides, although absolute numerical values for connectivity could be different, it can be expected that the same problems persist in the three groups analyzed (e.g., volume conduction, change in coupling), therefore the relative synchronization between them would remain the same. However, the use of metrics more adequate to measure functional connectivity, such as coherence, mutual information, phase synchronization, or Granger’s causality [28,29,30,31], would permit us to definitively solve this aspect.

It has been described that some patients with COVID-19 have symptoms similar to epilepsy [3] or sharp waves on the EEG [4]. However, in our series the presence of irritative activity was scarce. Probably the differential diagnosis search for confusion and cognitive complaints biased the diagnosis of epilepsy. Nonetheless, in the eight patients who showed a stuporous state, non-convulsive status epilepticus was suspected and finally discarded.

Although the features described for the three groups, including COVID, our main target, are robust enough to show statistically significant differences, obviously we need to increase the number of patients to attain a sharper and more exact definition of this entity. We cannot exclude that COVID patients express other EEG patterns and obviously a greater population will define better all the clinical spectrum of COVID encephalopathy. Additionally, we need to check whether COVID patients not admitted to the ICU but with cognitive complaints share the same EEG pattern.

## 5. Conclusions

Some severely affected COVID patients develop an encephalopathy with specific EEG features, with spectral and connectivity alterations, and raw tracings appear nearly physiological.

## 6. Patents

The numerical method used in this work is being evaluated for patent: Multivariate analysis method in EEG. Application number: P201930036. Application date: 01/21/2019. Pastor, J; Vega-Zelaya, L.

## Figures and Tables

**Table 1:** Comparison by paired groups of patients of EEG bands using $\chi^{2}$. Statistical significance for 5 degrees of freedom is indicated by *p* in the column to the right of every band.

Paired Groups	*δ*	*p*	θ	*p*	*α*	*p*	*β*	*p*
ENC/COVID	10.0	n.s	6.0	n.s	11.5	0.05	11.6	0.05
ENC/CRA	167.8	0.001	80.2	0.001	49.6	0.001	72.4	0.001
COVID/CRA	68.5	0.001	54.6	0.001	22.1	0.001	47.2	0.001

n.s., not significant.

**Table 2:** Comparison by paired groups of patients for Shannon’s spectral entropy (SSE) and *ρ* using $\chi^{2}$. Statistical significance for either 5 (SSE) or 7 (*ρ*) degrees of freedom (ν) is indicated by *p* in column to the right of every variable.

Paired Groups	SSE $\left(\chi^{2}\right)$	*p* (ν = 5)	$\rho \;(\chi^{2})$	*p* (ν = 7)
ENC/COVID	35.4	0.001	10.7	n.s
ENC/CRA	3.3	n.s	1.8	n.s
COVID/CRA	15.6	0.01	5.5	n.s

n.s., not significant.

## Appendix A

Clinical features of the groups of patients.

**Table A1:** Patients with infectious toxic encephalopathy (ENC).

Patient	Sex	Age (Years)	Medical History	Level of Awareness	EEG Findings
1	F	84		Stuporous	II
2	F	90	CD, HBP, DM, CLD	Stuporous	II
3	F	84	HBP, DM	Stuporous	III
4	F	72	HBP, CLD	Comatose	III
5	F	79	HBP	Confused	I
6	F	78	HBP	Fully alert	III
7	F	83	CD	Confused	II
8	M	71	CD, HBP	Confused	III
9	F	84	CD	Confused	III
10	M	42	ACLD	Comatose	III
11	F	88	CD, HBP, DM, CKD	Confused	III
12	M	53		Fully alert	II
13	M	80	CD, HBP	Fully alert	I
14	F	93	CD, HBP, CKD	Confused	III
15	F	86		Fully alert	II
16	M	82	CD, DM, CKD	Fully alert	II
17	M	81	CD, HBP, DM, CKD	Fully alert	III
18	M	82	CD, HBP, DM, CLD, I	Confused	II
19	F	92	CD, HBP	Stuporous	III
20	M	76	CD, HBP, DM, CKD, CLD	Comatose	III
21	M	18		Stuporous	III
22	F	56		Stuporous	III
23	M	83	CKD	Stuporous	III
24	M	83	HBP, DM	Confused	I
25	F	91	HBP	Stuporous	III
26	F	52		Comatose	IV
27	M	65		Fully alert	I
28	M	68	ACLD	Confused	II
29	M	77	CD, HBP, DM, CLD	Confused	III
30	M	56	CD, HBP, ACLD, CLD	Confused	II
31	M	70		Confused	II

ACLD, advanced chronic liver disease; CD, cardiovascular disease; CKD, chronic kidney disease; CLD, chronic lung disease; DM, diabetes mellitus; F, female; HBP, high blood pressure; I, immunosuppression; M, male. I–VI: grades of encephalopathy.

**Table A2:** Patients with SARS-CoV-2 (COVID).

Patient	Sex	Age (Years)	Medical History	Level of Awareness	SaO_2_ (%) (Mean ± SEM)/Minimum		EEG Findings
1	M	69	CD, HBP	Confused	92.3 ± 0.9	80	II, PLD
2	M	64		Confused	98.8 ± 0.4	91	I
3	M	36	CD, HBP, DM	Confused	97.2 ± 0.5	90	I
4	M	60	CD, HBP, DM, CLD	Stuporous	97.4 ± 0.5	89	II
5	M	61		Stuporous	95.1 ± 0.7	82	III
6	M	46		Confused	96.7 ± 0.5	83	III, SW
7	M	76	HBP	Confused	97.0 ± 0.7	80	I
8	F	74	CD, HBP	Fully alert	98.8 ± 0.6	88	II
9	M	60	CD, HBP, CLD	Stuporous	97.7 ± 0.4	88	III
10	M	55		Confused	95.6 ± 0.2	86	I, SW
11	M	61		Confused	91.2 ± 1.3	89	III
12	M	97	CD, HBP, CKD	Confused	95.0 ± 1.2	85	III, SW
13	M	63	ACLD	Confused	95.2 ± 0.6	91	II
14	M	61	CD, HBP, DM	Fully alert	96.2 ± 0.8	85	I
15	M	60		Stuporous	89.6 ± 1.2	81	III
16	F	71		Stuporous	91.2 ± 1.1	78	III
17	M	70	DM	Stuporous	91.5 ± 1.0	82	III
18	M	60	CD, HBP	Confused	94.6 ± 1.6	91	II
19	M	62	HBP	Stuporous	91.6 ± 2.2	89	III
20	F	72		Stuporous	93.6 ± 1.8	91	III

CD, cardiovascular disease; CLD, chronic lung disease; DM, diabetes mellitus; F, female; HBP, high blood pressure; M, male; PLD, periodic lateralized discharge; SW, sharp waves. I–VI: grades of encephalopathy.

**Table A3:** Patients with encephalopathy after cardiorespiratory arrest (CRA).

Patient	Sex	Age (Years)	Medical History	Level of Awareness	EEG Findings
1	M	63	CD, DM	Comatose	IV
2	M	60	CD, HBP	Stuporous	IV
3	M	56		Comatose	III
4	M	68	CD, HBP, CLD	Comatose	III
5	M	59	CD	Comatose	IV
6	M	87	CD, HBP, DM	Confused	III
7	M	76	CD, HBP, CKD	Comatose	IV
8	M	85	CD, HBP	Comatose	III
9	M	65	DM	Comatose	IV
10	F	63	CD, HBP, CLD	Confused	II
11	M	48	CD	Comatose	IV
12	M	49	CD, HBP, DM	Comatose	III
13	M	58	CD, DM, ACLD	Comatose	III
14	M	59	CD, HBP, DM, CLD	Comatose	IV
15	M	59		Comatose	IV
16	M	66	CD	Comatose	IV
17	M	71	CD, HBP, DM, CKD	Comatose	IV
18	M	59	CD	Confused	II
19	M	48	CD, HBP, CKD	Comatose	IV
20	M	45	DM, ACLD	Comatose	IV
21	M	75	CD, HBP, DM, CKD	Comatose	IV

ACLD, advanced chronic liver disease; CD, cardiovascular disease; CKD, chronic kidney disease; CLD, chronic lung disease; DM, diabetes mellitus; F, female; HBP, high blood pressure; M, male. I–VI: grades of encephalopathy.

## Appendix B

Algorithm used for qEEG

1. EEG channels were digitally filtered by a sixth-order Butterworth digital filter between 0.5 and 30 Hz.
2. Differential EEG montage was reconstructed. Topographic placement of channels was defined on the scalp as the midpoint between the electrode pairs defining the channel, e.g., the Fp_1_–F_3_ channel would be placed at the midpoint of the geodesic between Fp_1_ and F_3_ electrodes.
3. All recording was divided into 1 s moving windows with 10% overlap. This allowed us to use a precise frequency of 0.5 Hz. For each window (*n*) and frequency (*k*), we computed the fast Fourier transform (FFT) of the voltage ($V^{m}\left(n\right)$) obtained from every channel (m) to obtain the power spectrum ($S_{n,k}^{m}$, in µV^2^/Hz). We used this expression:
  (A1) $$S_{n,k}^{m}=\overset{N-1}{\underset{n=0}{\sum }}V^{m}\left(n\right)e^{-i\frac{2\pi }{N}kn};m=Fp_{1},\;F_{3},\ldots$$
  We also computed Shannon’s spectral entropy (SSE) according to:
  (A2) $$Se_{k}^{m}=-\overset{F}{\underset{k=0}{\sum }}p_{k}\log_{2}p_{k}$$
  where *F* is the maximum frequency computed and *p_k_* is the probability density of *S*, obtained from the expression:
  (A3) $$p_{k}=\frac{S_{n,k}^{m}}{\sum_{k=0}^{F}S_{n,k}^{m}\Delta k}$$
4. We computed the area under the $S_{n,k}^{m}$ according to the classical segmentation of EEG bands. We used this expression:
  (A4) $$A_{j}\left(k\right)=\overset{sup}{\underset{k=inf}{\sum }}\;S_{n}^{m}\left(k\right)\Delta k;j=\delta ,\;\theta ,\alpha ,\beta$$
  The expression *sup* refers to the upper limit of every EEG band.
5. The absolute value of Pearson’s correlation coefficient (ρ) was computed for every pair of channels (*i,j*) according to this expression:
  (A5) $$\rho_{ij}^{k}=\frac{\sum_{k=1}^{N_{window}}\left(x_{i}\left(k\right)-{\bar{x}}_{i}\right)\sum_{k=1}^{N_{window}}\left(x_{j}\left(k\right)-{\bar{x}}_{j}\right)}{\sqrt{\sum_{k=1}^{N_{window}}{\left(x_{i}\left(k\right)-{\bar{x}}_{i}\right)}^{2}\sum_{k=1}^{N_{window}}{\left(x_{j}\left(k\right)-{\bar{x}}_{j}\right)}^{2}}}$$
  where N_window_ is the number of points included in a window (usually 128) and ${\bar{x}}_{i},{\bar{x}}_{j}$ represents the mean of both channels.
6. The mean value of all windows was computed, obtaining the mean correlation matrix.

Areas of the same band were grouped by cerebral lobe. In the case of the left hemisphere (shown as an example), we grouped frontal $F=\left\{\frac{\left(Fp_{1}-F_{3}\right)+\left(F_{3}-C_{3}\right)+\left(Fp_{1}-F_{7}\right)}{3}\right\}$, parieto-occipital $PO=\left\{\frac{\left(C_{3}-P_{3}\right)+\left(P_{3}-O_{1}\right)+\left(T_{5}-O_{1}\right)}{3}\right\}$, and temporal $T=\left\{\frac{\left(Fp_{1}-F_{7}\right)+\left(F_{7}-T_{3}\right)+\left(T_{3}-T_{5}\right)+\left(T_{5}-O_{1}\right)}{4}\right\}$. Channels from the right hemisphere were grouped accordingly. These areas, for both bands (*j*) and lobes (*r*), $A_{j}^{r}\left(t\right);\;r=F,\;PO,\;T$, were plotted as time functions and compared between hemispheres. The same groups were used to compute SSE.
